# Pathophysiological mechanisms, diagnostic innovations, and multimodal therapeutic strategies for slow transit constipation

**DOI:** 10.1186/s12876-025-04387-9

**Published:** 2025-11-17

**Authors:** Xuesong Tang, Yiman Huang, Tao Jiang, Jiaxin Wu, Keying Wang, Wenjiang Wu

**Affiliations:** Department of Anorectal, Shenzhen Hospital (Futian) of Guangzhou University of Chinese Medicine, Shenzhen, China

**Keywords:** Slow transit constipation, Colonic motility, Interstitial cells of Cajal, Rome criteria, Prucalopride, Colectomy

## Abstract

**Background:**

Slow transit constipation (STC), a subtype of functional constipation characterized by delayed colonic transit (> 72 h), imposes substantial physical and psychological burdens.

**Aims:**

This review synthesizes current evidence on STC epidemiology, pathophysiology, diagnostic innovations, and multimodal management.

**Methods:**

A literature review was conducted using PubMed and China National Knowledge Infrastructure (CNKI) for all articles and trials with the following search terms: "Slow transit constipation" OR "Chronic constipation" OR "STC". The comprehensive literature search was conducted for relevant articles published up to June 2025. The search yielded approximately 1005 records from PubMed and 220 records from CNKI. The abstracts and titles of all retrieved articles were reviewed for relevance. Articles were included in this narrative review if they provided original insights, comprehensive summaries, or presented clinical trial data on the pathophysiology, diagnostic innovations, or therapeutic strategies for slow transit constipation. Both foundational and recent high-impact studies were prioritized.

**Results:**

Key pathophysiological mechanisms involve: smooth muscle atrophy/fibrosis, interstitial cells of Cajal (ICC) depletion, enteric nervous system dysregulation, hormonal imbalances (thyroid/sex hormones), and gut microbiota dysbiosis. Diagnosis integrates: (1) Rome IV symptom criteria (≤ 3 spontaneous bowel movements/week, hard stools, straining); (2) Objective transit testing (scintigraphy, wireless motility capsule); (3) Exclusion of secondary causes. Conservative management emphasizes fiber intake, hydration, and physical activity. Pharmacotherapy includes osmotic laxatives (first-line), prokinetics (prucalopride; second-line), and microbiota modulators. For refractory cases, colectomy requires strict selection: failed conservative therapy, confirmed transit delay, and excluded pelvic floor dysfunction. Future research should prioritize genetic susceptibility, signaling pathway modulation, and personalized algorithms.

**Conclusions:**

The explicit pathogenesis of STC remains incompletely characterized. Besides referencing the Rome IV criteria, its diagnosis requires a comprehensive assessment integrating the clinical symptoms and various accessorial examinations. The management of STC also demands clinical gastroenterologist adopt a comprehensive approach, including appropriate lifestyle, pharmacological interventions, psychotherapy, and even surgical treatment. This review provides multidimensional ideas for gastroenterologists to treat patients grappling with STC.

## Introduction

In contemporary society, constipation has emerged as a prevalent gastrointestinal disorder, significantly impairing patients’ quality of life. Etiologically, chronic constipation is classified into primary and secondary forms [1], with functional constipation representing the predominant subtype of primary constipation. Functional constipation can be further divided into normal transit constipation, slow transit constipation (STC), and defecatory disorders [2]. Although STC does not represent the most prevalent subtype of functional constipation, its complex pathophysiological mechanisms and the considerable challenges associated with clinical management have increasingly attracted attention within the medical community in recent years. Due to its protracted course, therapeutic challenges, and refractory symptoms, slow transit constipation (STC) exerts a profound impact on patients’ physical health while imposing substantial economic and psychological burdens [3, 4]. Given the complex pathophysiology of STC and its profound impact on patient quality of life and healthcare resources, advancing our understanding of its underlying mechanisms, refining diagnostic criteria, and optimizing therapeutic algorithms are of significant clinical and societal value.

This study aims to elucidate the pathophysiological mechanisms, refine diagnostic accuracy, and optimize therapeutic strategies for slow transit constipation (STC). Through a comprehensive synthesis of current literature and integration of clinical experience, the investigation seeks to identify underlying etiological factors contributing to STC, establish more sensitive and specific biomarkers for early diagnosis, and enhance treatment protocols to improve patient quality of life. Furthermore, this study holds substantial significance for advancing the understanding of STC and fostering the development of the field.

## Epidemiological characteristics

### Global variability in prevalence

Current epidemiological data on STC remain limited, with most available evidence derived from broader studies of functional constipation (FC). The prevalence of FC demonstrates significant geographic heterogeneity, likely attributable to ethnic differences, environmental exposures, dietary patterns, lifestyle factors, and inconsistencies in diagnostic criteria and research methodologies. A meta-analysis study in 2021 based on the Rome Criteria reported a global prevalence of functional constipation ranging from 10.1% to 15.3% [5]. Other studies have found that functional constipation is mainly concentrated in women [6], people with little activity [7, 8], the elderly [9] and people with lower socioeconomic status [10, 11]. STC, representing the least common FC subtype, accounts for approximately 15%–30% of FC cases [12]. However, its true prevalence may be underestimated due to the requirement for specialized colonic transit studies for diagnosis. These epidemiological features underscore the necessity for enhanced screening and early identification of high-risk populations in clinical practice.

A 2020 Rome Foundation [13] survey of 73,076 adults across 33 countries revealed that European nations such as France, Italy, Poland, and Turkey exhibit relatively high FC prevalence rates (14.1%–14.5%). In the United States, FC prevalence is approximately 8.7%. A 2021 study by Tanner et al. reported that 55% of constipated Americans met criteria for STC [14]. These prevalence rates are closely linked to regional dietary habits; Western populations typically consume diets high in protein and fat but low in dietary fiber, contributing to reduced colonic motility, harder stools, and increased STC risk. Additionally, fast-paced lifestyles, chronic psychological stress, and insufficient physical activity further impair gastrointestinal motility, promoting the development of STC.

In Asia, Japan reports the highest prevalence of chronic constipation at 16.6%, a figure associated with traditional dietary patterns (refined grains, seafood, low fiber intake), increasing work-related stress, and sedentary behaviors. A 2021 Japanese study indicated that the actual prevalence of constipation may be underestimated due to the stringent Rome IV criteria, which exclude some patients with clinically significant symptoms [7].

In developing countries, STC prevalence is highly variable, influenced by dietary composition, occupational activity, lifestyle, and educational attainment. Regions with high consumption of fiber-rich foods and greater physical labor report lower STC rates. However, globalization has led to increased intake of high-calorie, low-fiber foods and reduced physical activity, resulting in a rising STC prevalence. A study on chronic constipation in China reported an overall adult prevalence of 10.9%, with higher rates in females, northern regions, older age groups, and individuals with higher educational attainment, highlighting the multifactorial influences of age, sex, geography, and education [15].

### Population distribution characteristics

STC exhibits distinct distribution patterns across demographic groups, with age, sex, and occupation significantly influencing disease prevalence.

#### Age-related analysis

STC prevalence increases with advancing age. In pediatric populations, STC is relatively uncommon and is primarily associated with formula feeding, dietary transitions, fiber intake, and genetic factors [16]. Recent research implicates deficiencies in substance P, vasoactive intestinal peptide (VIP), and abnormalities in interstitial cells of Cajal as contributors to enteric neuronal dysplasia in pediatric STC [17].

Young and middle-aged adults, particularly women aged 30–40 years, represent a high-risk group [2]. Accelerated lifestyles, occupational stress, chronic psychological tension, and irregular eating patterns disrupt neuroendocrine homeostasis and impair colonic motility. In addition, hormonal fluctuations and mechanical effects during pregnancy and postpartum periods further predispose middle-aged women to STC.

In the elderly, STC prevalence rises again, driven by dietary changes, reduced physical activity, polypharmacy for comorbidities [18], and intrinsic factors such as slowed colonic transit, pelvic floor dysfunction, and muscular atrophy [9].

#### Sex differences

Females are approximately twice as likely as males to develop STC [19], a disparity linked to unique physiological and psychological factors. Constipation affects up to 40% of pregnant women [20], with elevated progesterone levels, overexpression of progesterone receptors, downregulation of stimulatory G proteins, and upregulation of inhibitory G proteins prolonging colonic transit time and fecal retention [21]. Vaginal delivery may cause pelvic floor and neural injury, further impairing defecatory function. Hormonal fluctuations during the menstrual cycle can also precipitate gastrointestinal dysmotility and constipation.

Psychological factors exert a greater influence on STC in females, as women are more susceptible to mood disturbances. Chronic anxiety and depression disrupt neurotransmitter secretion (e.g., serotonin, dopamine), which are integral to colonic motility regulation, thereby prolonging colonic transit and increasing STC risk [22].

#### Occupational influences

Although occupation is not a direct etiological factor, occupational characteristics significantly impact lifestyle habits and, consequently, STC prevalence. Sedentary workers, such as office employees, experience reduced gastrointestinal stimulation and slower colonic transit. High-stress occupations induce autonomic dysfunction, exacerbating gastrointestinal dysmotility. Individuals with demanding schedules may have irregular eating and hydration patterns and insufficient fiber intake, predisposing to STC. Conversely, excessive physical labor may lead to fatigue and impaired digestive function, further hindering colonic transit and promoting STC.

## Pathophysiological mechanisms

**Fig. 1:**
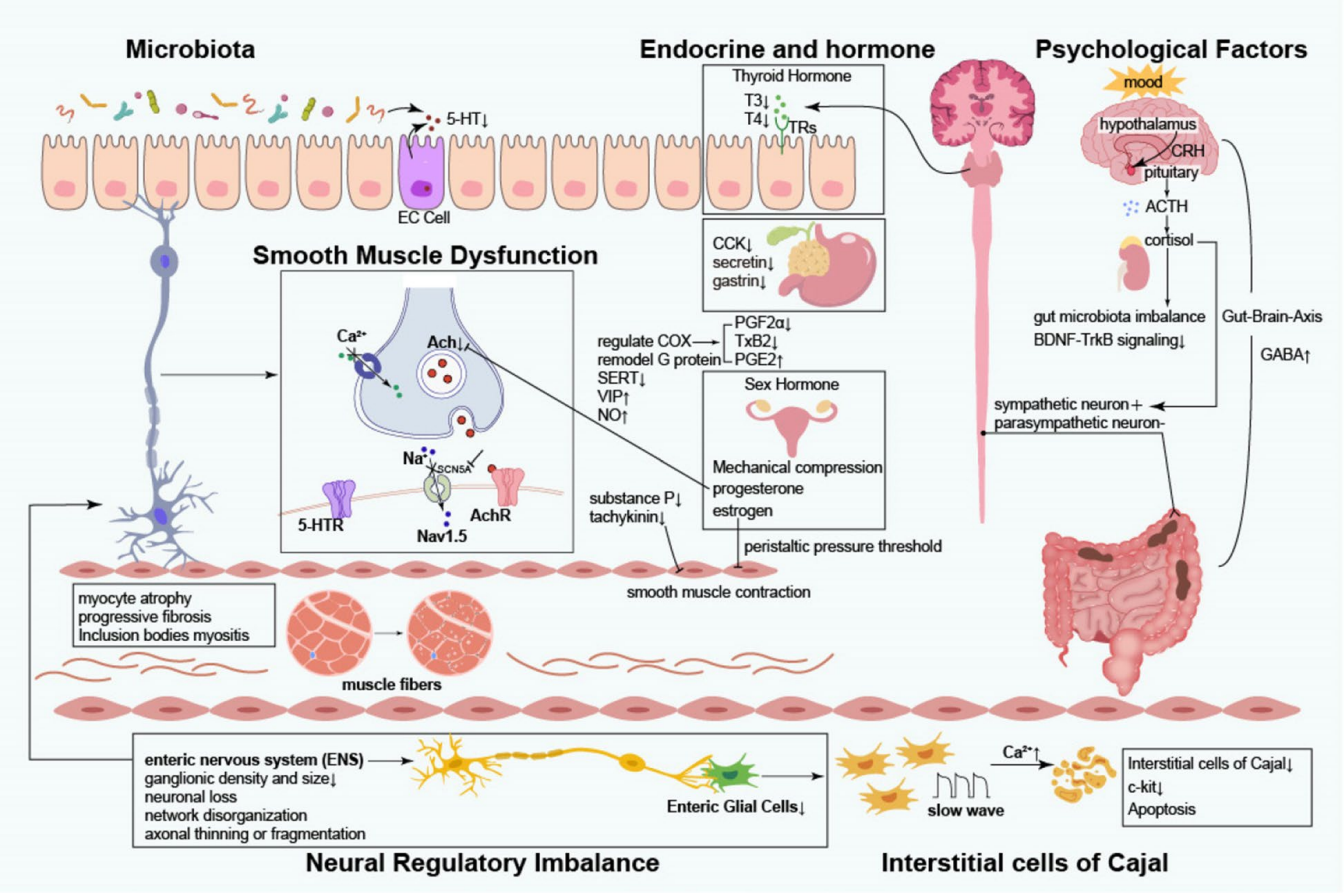
Proposed pathophysiology of Slow Transit Constipation. 5-HT: 5-Hydroxytryptamine, serotonin; 5-HTR: 5-Hydroxytryptamine receptor; CCK: Cholecystokinin; EC Cell: Enterochromaffin (EC) cell; T3: Triiodothyronine; T4: Thyroxine; CRH: corticotropin releasing hormone; ACTH: adrenocorticotropic-hormone; GABA: Gamma amino butyric acid; Ach: acetylcholine; AchR: acetylcholine receptor; NO: nitricoxide; VIP: vasoactive intestinal peptide; SERT: Serotonin Transporter; PGF2α: Dinoprost; TxB2: thromboxane B2; PGE2: Prostaglandin E2; BDNF: brain-derived neurotrophic factor; TrkB: tyrosine kinase receptor B

From a pathophysiological perspective, in-depth exploration of colonic dysmotility, neuroendocrine regulatory imbalances, and alterations in the intestinal microbiota will facilitate the development of targeted pharmacological interventions and novel therapeutic modalities (Fig. 1).

### Etiology of colonic dysmotility

Colonic dysmotility constitutes the central pathophysiological process in slow transit constipation (STC), involving multifactorial dysfunction across intestinal smooth muscle, interstitial cells of Cajal (ICC), neural transmission, hormonal milieu, and psychological factors. These elements interact synergistically, culminating in impaired colonic transit.

#### Smooth muscle dysfunction

The coordinated contraction and relaxation of intestinal smooth muscle underpin normal peristalsis and fecal propulsion. In STC, characteristic pathological alterations are observed within the smooth muscle apparatus: (1) myocyte atrophy, evidenced by reduced fiber diameter and downregulation of contractile protein expression, resulting in diminished contractile force; (2) progressive fibrosis, marked by increased collagen deposition and muscularis thickening, severely restricting smooth muscle compliance. The pathogenesis of these changes is multifactorial: chronic constipation-induced intraluminal hypertension disrupts myocyte metabolism and protein synthesis; persistent inflammatory responses stimulate fibroblast proliferation and excessive collagen secretion, leading to perimuscular fibrosis and impaired extensibility [23]. Additionally, a subset of STC patients exhibit inclusion body myopathy [24], a secondary myopathic injury due to denervation or fecal stasis, further exacerbating dysmotility. Collectively, these structural abnormalities form the anatomical substrate for impaired colonic transit in STC and represent critical therapeutic targets.

From a molecular pathology perspective, dysregulation of the ion channel regulatory network in colonic smooth muscle cells represents a key mechanism underlying intestinal motility disorders in slow transit constipation (STC) [25]. Calcium ions serve as pivotal second messengers in the regulation of smooth muscle contraction [26], and disruption of their homeostasis significantly impacts gastrointestinal function and defecation processes [27]. In STC patients, reduced expression or impaired function of calcium channel proteins on intestinal smooth muscle cells hinders calcium influx, thereby failing to effectively initiate smooth muscle contraction. Additionally, diminished capacity of intracellular calcium stores, such as the endoplasmic reticulum, to release calcium further compromises contractile force [28]. Notably, Mazzone [29] and colleagues identified distinct microRNA (miRNA) expression profiles in the colonic tissue of STC patients, with four specific miRNAs capable of targeting and suppressing the SCN5A gene, which encodes the voltage-gated sodium channel NaV1.5. This modulation affects the generation and propagation of action potentials in smooth muscle cells. The synergistic dysfunction of these ion channels ultimately results in reduced amplitude and delayed propagation of colonic peristaltic waves, forming the characteristic pathophysiological basis of STC.

#### Interstitial Cells of Cajal (ICC)

ICC serve as the gastrointestinal pacemaker cells, orchestrating rhythmic slow-wave potentials that drive phasic smooth muscle contractions [30, 31], as first described by Santiago Ramón y Cajal in 1911 [32]. These cells generate periodic depolarizations, which, via electromechanical coupling, induce coordinated peristalsis [33]. Recent studies demonstrate a marked reduction in ICC density in STC colonic tissue [34–37], accompanied by downregulation of c-kit gene transcription and protein expression [38]. These findings implicate c-kit pathway dysfunction and ICC depletion or impairment as central to STC pathogenesis. Furthermore, experimental models reveal that elevated intracellular Ca2 + in ICC may trigger autophagic apoptosis, precipitating STC [39].

#### Neural regulatory imbalance

Normal intestinal motility is governed by an intricate neural network, encompassing the enteric nervous system (ENS), central nervous system (CNS), and autonomic nervous system (ANS). The ENS, often termed the “second brain” of the gut [40], independently regulates motility, secretion, and local blood flow, while integrating with the CNS via sympathetic ganglia and the gut-pancreatic endocrine axis [41]. In STC, the ENS exhibits degenerative changes, including reduced myenteric plexus ganglion density, neuronal loss, network disorganization, and axonal thinning or fragmentation [42], resulting in impaired neuromuscular coordination. Neurotransmitter imbalance is also evident in STC. Diminished cholinergic excitatory input and increased non-adrenergic, non-cholinergic inhibitory signaling contribute to colonic hypomotility. The secretion of excitatory neurotransmitters such as acetylcholine [43], substance P [44, 45], tachykinin and serotonin(5-HT) [46] is reduced, which weakens the stimulation signal for smooth muscle contraction. At the same time, inhibitory neurotransmitters such as vasoactive intestinal peptide (VIP) [47, 48], nitric oxide (NO) [49] and Gamma-Aminobutyric Acid (GABA) [50] increased relatively, further inhibiting the contraction of smooth muscle, resulting in profound colonic stasis. Enteric glial cells (EGCs) are essential for maintaining neuronal microenvironmental homeostasis and synaptic function. EGC depletion in STC may exacerbate neuronal and ICC degeneration [51, 52], and inhibition of EGC apoptosis ameliorates STC symptoms [53], underscoring the importance of EGC dysfunction in disease pathogenesis. ANS dysfunction also contributes to STC [54]. Normally, sympathetic activity inhibits, while parasympathetic activity promotes, colonic motility [55]. In STC, excessive sympathetic activation and parasympathetic degeneration [56] impede peristaltic initiation, aggravating constipation.

Thus, ENS structural abnormalities, neurotransmitter imbalance, EGC dysfunction, and ANS dysregulation collectively underpin colonic dysmotility in STC.

### Endocrine and hormonal factors

Dysregulation of endocrine and hormonal pathways plays a significant role in STC pathogenesis. Studies indicate that STC patients exhibit marked dysfunction of intestinal endocrine cells, including reduced numbers of enteroglucagon and serotonin (5-hydroxytryptamine) cells [57], and decreased secretory indices for enteroglucagon and somatostatin.

These deficits may attenuate colonic motility and promote STC progression. Alterations in systemic hormones such as thyroxine and progesterone are also closely linked to STC.

#### Thyroid hormone effects

Thyroid hormones, critical regulators of basal metabolic rate, are intimately associated with STC [19]. Physiologically, thyroxine (T4) and triiodothyronine (T3) bind to intracellular thyroid hormone receptors (TRs) in intestinal epithelial cells, modulating metabolic activity, proliferation, and differentiation to sustain normal motility. Hypothyroidism impairs hormone synthesis, leading to myocyte metabolic dysfunction, reduced contractility, delayed epithelial turnover, compromised nutrient absorption, and ENS impairment, collectively resulting in delayed colonic transit and fecal desiccation characteristic of STC [58].

Notably, approximately 50% of primary hyperparathyroidism (PHPT) patients experience constipation, which improves post-parathyroidectomy [59], suggesting that calcium-phosphate metabolic disturbances may influence neuromuscular excitability and STC pathogenesis.

#### Sex hormone associations

Sex hormones are pivotal in female STC pathophysiology. Fluctuations in estrogen and progesterone during menstruation, pregnancy, and menopause correlate with STC incidence and severity. Estrogen elevates the peristaltic pressure threshold, suppressing smooth muscle activity and predisposing to STC [60–62]. Progesterone exerts multifaceted effects: inhibiting acetylcholine release, promoting NO synthesis, and modulating ICC function, thereby relaxing smooth muscle and retarding transit [63, 64]. Animal studies confirm that high progesterone with low estradiol significantly reduces gastrointestinal contractility and delays gastric emptying [65]. Clinical monitoring reveals prolonged colonic transit and exacerbated constipation during the luteal phase, coinciding with elevated progesterone [66]. During pregnancy, high progesterone levels downregulate SERT, increase 5-HT (without prokinetic effect due to impaired muscle contractility), and alter cyclooxygenase (COX) isoform expression, reducing contractile prostaglandins and increasing relaxant prostaglandins, thereby impairing colonic contractility and basal motility [67–69]. Progesterone also remodels G protein subunit expression and mediates VIP-dependent inhibition of muscle contraction [21, 70]. Mechanical compression by the gravid uterus further impedes transit, explaining the high prevalence of STC in pregnancy. Postmenopausal hormonal decline, while not directly affecting transit [71, 72], contributes to sarcopenia and muscle atrophy, impairing colonic function and increasing STC risk with advancing age [73–75]. Perimenopausal stress and anxiety further exacerbate STC [76].

#### Cholecystokinin (CCK), gastrin, and secretin

Elevated fasting and postprandial plasma levels of CCK, gastrin, and secretin have been documented in STC, particularly in patients with severe transit impairment. These gastrointestinal hormones may modulate colonic transit and retard motility, although their precise molecular mechanisms remain to be elucidated [77–79].

### Psychological factors

Psychological factors are integral to STC pathogenesis, mediated via complex neuroendocrine-immune networks. Chronic affective disorders (anxiety, depression) and sustained psychological stress activate the hypothalamic–pituitary–adrenal (HPA) axis and disrupt autonomic function, significantly impairing colonic motility and secretion.

#### Stress response mechanisms

Acute or chronic stress rapidly activates the HPA axis [80, 81], with hypothalamic corticotropin-releasing hormone (CRH) stimulating pituitary adrenocorticotropic hormone (ACTH) release, which in turn promotes adrenal glucocorticoid (cortisol) secretion [82, 83]. Cortisol mobilizes energy reserves and modulates mood [84], but chronic elevation, in concert with sympathetic activation (via norepinephrine release) [85], acts on β-adrenergic receptors to suppress smooth muscle contraction and delay transit, precipitating STC.

Stress-induced gut dysfunction involves: (1) a vicious cycle between cortisol dysregulation and gut microbiota imbalance, exacerbating dysfunction [86]; (2) The neurotrophic factor system, comprising brain-derived neurotrophic factor (BDNF) and its receptor tropomyosin receptor kinase B (TrkB), modulates neuronal signaling within the central nervous system (CNS) and directly influences extrinsic muscle excitatory potentials, thereby facilitating gastrointestinal motility. Studies have demonstrated that stress not only induces significant alterations in neuronal function within the CNS, resulting in marked and observable behavioral changes, but also markedly suppresses BDNF expression and attenuates BDNF-TrkB signaling. This suppression leads to delayed intestinal peristalsis, contributing to the development of slow transit constipation (STC) [87, 88]; (3) bidirectional modulation of the gut-brain axis by stress-related neurotransmitters and hormones, further aggravating dysmotility.

#### Impact of chronic psychological states

Persistent negative affective states adversely affect the gut microenvironment and neural pathways [89], explaining the higher STC prevalence in patients with anxiety and depression [90]. At the microbiome level, these states alter microbial composition and diversity; for example, overgrowth of Bacteroides in depression degrades mucins, thins the mucus barrier, impairs gut integrity, and increases GABA production [50], thereby slowing motility [91]. Neurotransmitter dysregulation along the gut-brain axis, particularly elevated CRF, further increases STC risk [92, 93]. Integrative omics analyses confirm a molecular link between depressive symptoms and constipation [94].

### Gut microbiota dysbiosis

The human gut harbors a complex microbial ecosystem intimately linked to health and disease [95]. Gut microbiota significantly promote 5-HT biosynthesis [96, 97], primarily via enterochromaffin (EC) cells and tryptophan hydroxylase-1 (TPH1) activity [98]. As a key neurotransmitter and paracrine signal, 5-HT directly stimulates smooth muscle contraction and modulates ENS neuronal activity, thus maintaining normal defecation [99]. Microbial dysbiosis disrupts 5-HT synthesis, release, and reuptake, leading to 5-HT imbalance, impaired colonic transit, and ultimately, the development of STC.

## Diagnostic methods and technological advances

**Fig. 2:**
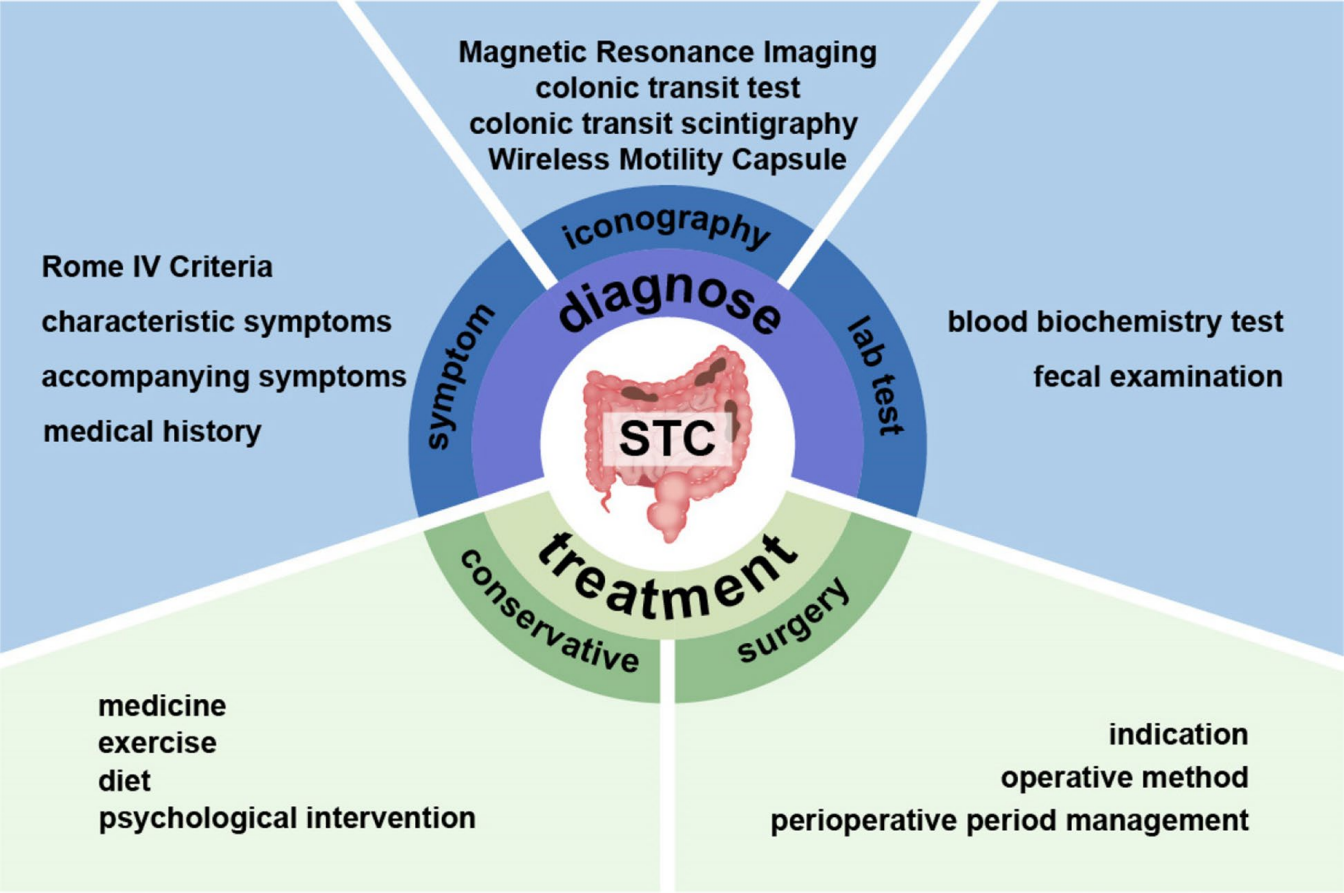
The diagnose and treatment of STC. STC, Slow Transit Constipation

Accurate diagnosis is essential to minimize misdiagnosis and missed diagnoses, enabling the formulation of individualized treatment regimens and reducing unnecessary therapeutic risks and resource expenditure (Fig. 2).

### Key points in clinical symptom assessment

Clinical symptom assessment constitutes a pivotal component in the diagnostic algorithm for slow transit constipation (STC). In current clinical practice, the diagnosis of STC is primarily guided by the diagnostic framework for functional constipation as delineated in the Rome III or Rome IV criteria. However, it is critical to note that these criteria do not adequately capture the pathognomonic feature of STC—namely, prolonged colonic transit time. Therefore, the clinical diagnostic process should emphasize: (1) identification of characteristic symptoms, with particular attention to those indicative of colonic dysmotility; (2) interpretation of ancillary test results, especially objective evidence from colonic transit studies; and (3) comprehensive history-taking to elucidate disease course and associated symptoms [100]. This multidimensional assessment paradigm is essential for the accurate diagnosis of STC and for distinguishing it from other constipation subtypes, such as defecatory outlet obstruction.

#### Analysis of typical symptoms

The most salient clinical feature of STC is a marked reduction in defecation frequency. Epidemiological data indicate that healthy individuals typically maintain a “3 + 3” bowel habit (no more than three times daily, at least three times weekly, i.e., > 21 times per month) [101], whereas most STC patients fall below the threshold of three bowel movements per week. Notably, clinical observations reveal that a defecation frequency of approximately 5.6 times per week may still be consistent with an STC diagnosis, underscoring the heterogeneity of the disorder [102]. This decline in frequency directly reflects severe impairment of colonic transit: prolonged intraluminal stool retention (usually > 72 h) leads to excessive water reabsorption and the formation of desiccated, hard stools. Concurrently, the accumulation of luminal contents results in the retention of deleterious metabolic byproducts, which, via the gut-brain axis, further suppress colonic motility and perpetuate a progressive vicious cycle. This pathophysiological cascade not only alters stool consistency Bristol Stool Form Scale types 1–2 (hard, lumpy stools) but also constitutes the mechanistic substrate for the clinical manifestations of STC.

Fecal desiccation in STC patients exhibits distinctive pathological features and clinical significance. The underlying mechanism is primarily attributable to delayed colonic transit, resulting in prolonged stool retention (typically exceeding 72 h), during which continuous water absorption reduces fecal water content below critical levels, yielding characteristically hard, lumpy stools (Bristol types 1–2). Such pathological changes in stool consistency may precipitate a spectrum of complications [103, 104]: (1) mechanical injury (e.g., anal fissures, hemorrhoids); (2) neuroreflexive inhibition of defecation; and (3) psychogenic fear of defecation. These complications, through a pain-inhibition cycle, further exacerbate colonic dysfunction and significantly impair quality of life, thereby representing key therapeutic targets in the clinical management of STC.

Defecatory difficulty in STC is characterized by distinct pathophysiological features. The core manifestation is the need for prolonged straining and Valsalva maneuvers during defecation, often accompanied by compensatory abdominal muscle tension and autonomic symptoms (e.g., diaphoresis, facial flushing). This arises from ineffective colonic propulsive activity, necessitating increased intra-abdominal pressure to facilitate stool passage. Chronic aberrant defecatory patterns may induce pelvic floor muscle decompensation [105], manifesting as: (a) pelvic floor muscle fatigue; (b) rectoanal dyssynergia; and (c) sensations of incomplete evacuation and rectal fullness. This vicious cycle of dysmotility, structural alteration, and functional impairment is a critical driver of disease progression and a focal point for therapeutic intervention.

#### Analysis of associated symptoms

Abdominal distension is among the most prevalent concomitant symptoms in STC [106]. Its pathogenesis is primarily linked to colonic dysmotility, resulting in fecal stasis and bowel dilation, with specific mechanisms including: (1) delayed colonic transit and stool retention; (2) abnormal fermentation and gas production; and (3) heightened visceral sensitivity. This symptom often exhibits diurnal variation, worsening postprandially and in the evening, thereby adversely affecting appetite and daily functioning. Chronic distension may further precipitate dyspepsia and nausea, compounding the reduction in quality of life. The transition from motility disorder to functional disturbance constitutes a vicious cycle that not only intensifies patient suffering but also impacts therapeutic efficacy, warranting particular attention in clinical management.

Abdominal pain is also reported in a subset of STC patients, with variable characteristics including dull, distending, or colicky pain, most commonly localized to the lower abdomen or periumbilical region. The heterogeneity of pain reflects diverse pathophysiological mechanisms: (1) mechanical factors—bowel distension from fecal impaction stretches the enteric plexus, eliciting pain; (2) functional factors—spasmodic pain from abnormal smooth muscle contractions; and (3) altered neural sensitivity—visceral hypersensitivity amplifies nociceptive transmission. The frequency and severity of pain are patient-specific, ranging from occasional mild discomfort to frequent, severe episodes necessitating analgesic intervention. Importantly, pain severity often correlates positively with constipation chronicity, suggesting a role for chronic inflammation and neuroplastic changes in pain persistence. This complex symptomatology not only exacerbates patient distress but may also confound therapeutic outcome assessment, highlighting the need for careful clinical attention.

Nausea and vomiting are infrequent in early disease but may become prominent as STC progresses or with prolonged disease duration, particularly when massive fecal accumulation increases the risk of colonic obstruction. The underlying mechanism involves elevated intraluminal pressure stimulating gastrointestinal mechanoreceptors, which, via vagal reflex pathways, trigger emetic responses [107]. The emergence of such symptoms often signals a more severe disease stage, necessitating urgent medical evaluation and comprehensive management, including relief of obstruction and maintenance of fluid-electrolyte balance, to prevent catastrophic complications such as perforation or sepsis.

#### Relevant history taking and data collection

A multidimensional approach is required when evaluating clinical symptoms, encompassing presence, duration, severity, and dynamic evolution. (1) Symptom duration: Chronicity often indicates progressive functional impairment and may portend irreversible pathological changes. (2) Severity assessment: This informs disease staging and therapeutic prioritization. For instance, patients with extremely low defecation frequency (≤ 1/week), hard stools (Bristol types 1–2), and severe, frequent abdominal pain may warrant early surgical evaluation or intensified pharmacological intervention. (3) Dynamic trends: Symptom progression or amelioration is critical for therapeutic adjustment. Progressive worsening may indicate increased obstruction risk or failure of conservative therapy, necessitating prompt escalation of care; conversely, symptom improvement post-intervention may validate current management strategies.

Additionally, thorough inquiry into past medical history is essential, including prior abdominal surgeries, medication use, and comorbidities (e.g., hypothyroidism, diabetes mellitus, pheochromocytoma) [106, 108]. Thus, systematic and dynamic clinical assessment not only optimizes individualized treatment decisions but also provides a foundation for prognostic evaluation.

### Imaging modalities

Imaging studies play a central role in the diagnosis of STC, with a range of advanced modalities providing robust support for precise diagnosis, each with distinct advantages and limitations.

#### Colonic transit studies

Colonic transit studies [109–111] represent the standardized method for evaluating colonic motor function. Subjects ingest a specified number of radiopaque markers (e.g., barium or radioisotope-labeled particles), at a standardized time (typically in the morning of the test day), serial abdominal radiographs are then obtained at 24,48, and, if necessary, 72 h post-ingestion. Analysis of marker distribution across the ascending, transverse, descending, and rectosigmoid colon [112], combined with calculation of the colonic transit index, allows for precise assessment of global and segmental transit function. The underlying principle is that normal colonic transit is achieved through coordinated segmental contractions and propulsive peristalsis, whereas in STC, impaired motility (manifested as reduced amplitude and frequency of peristaltic waves and disordered coordination) results in significant marker retention, particularly in the right colon. This method not only objectively quantifies colonic transit but also accurately localizes sites of delayed transit, providing critical data for diagnosis and therapeutic planning.

This simple and inexpensive test provides an assessment of whole-gut transit time, exhibiting high specificity but only moderate sensitivity for diagnosing STC.

However, several technical limitations exist in the clinical application of colonic transit studies. First, in patients with anatomical variants such as redundant or tortuous colon, mechanical retention of markers in specific segments may yield false-positive results. Second, some patients may self-administer laxatives or enemas during the study due to intolerable symptoms, artificially accelerating marker expulsion and resulting in false-negative findings. To mitigate these confounders, repeated testing is often required to enhance result reliability, though this increases both radiation exposure and healthcare resource utilization. Consequently, clinical guidelines recommend at least two consistent positive results before using findings to guide management. These limitations underscore the necessity of integrating anatomical, clinical, and pharmacological data when interpreting test results.

#### Colonic scintigraphy

Colonic transit scintigraphy, as a safe and noninvasive functional assessment modality, enables simultaneous evaluation of proximal colonic emptying and overall colonic transit status [113, 114]. Currently, two principal scintigraphic protocols are employed in clinical practice: first, following an 8–12 h fast, subjects orally ingest a pH-sensitive methacrylate-coated capsule containing 111In-labeled activated charcoal particles, which dissolves in the alkaline environment of the terminal ileum to release the tracer [115]; second, 111In-DTPA-labeled liquid is directly instilled into the cecum via colonoscopy [106, 116], utilizing the transit properties of radiolabeled water to assess both small and large intestinal motility [117]. During the examination, subjects undergo anterior and posterior abdominal gamma camera scans (2 min per scan) at 4, 6, 8, 24, and 48 h post-tracer administration, with dynamic imaging used to analyze colonic transit function [118]. Notably, to reduce procedural costs, some centers have adopted 67 Ga-citrate as a more economical alternative tracer [119].

Quantitative analysis of the geometric center (GC) values of the radiotracer at various time points provides an objective assessment of colonic transit function. Diagnostic criteria are as follows: for female patients, a 24-h GC < 1.3 or 48-h GC < 1.9; for male patients, a 24-h GC < 1.5 or 48-h GC < 2.1. Additionally, a 48–24 h GC increment (Δ48–24) < 0.29 in males or < 0.38 in females indicates significantly impaired colonic transit, and, when combined with clinical manifestations, is diagnostic for slow transit constipation (STC). This quantitative standard demonstrates robust objectivity and reproducibility, providing a critical basis for the clinical diagnosis of STC [106].

As the gold-standard for quantifying regional colonic transit, it offers dynamic physiological data, demonstrating both high sensitivity and high specificity for detecting colonic dysmotility.

$$\begin{aligned}\mathrm{GC}=&\left\{\left[\left(\%\mathrm{AC}\times1\right)+\left(\%\mathrm{TC}\times2\right)\right.\right. \\& \left. \left. +\left(\%\mathrm{DC}\times3\right)+\left(\%\mathrm{RS}\times4\right)\right.\right. \\& \left. \left. +\left(\%\mathrm{stool}\times5\right)\right]\right\}/100\end{aligned}$$

$$\mathrm{Colonic}\;\mathrm{Transit}\;\mathrm{Index}\;(\mathrm{CTI})\;=\triangle\mathrm{GC}\left(48\mathrm h-24\mathrm h\right)$$

The limitations of this modality include the requirement for specialized nuclear medicine equipment and personnel, high cost, and procedural complexity.

#### Wireless motility

Capsule in Colonic Transit Assessment The wireless motility capsule (WMC) is a miniaturized device integrating pH, pressure, and temperature sensors, capable of accurately measuring transit times throughout the entire gastrointestinal tract (stomach, small intestine, and colon) [114]. The standard protocol requires discontinuation of all prokinetic and acid-suppressive medications one week prior to testing. On the day of examination, after an overnight fast, the subject consumes a 260 kcal standardized nutritional bar, followed by ingestion of the WMC with 200 mL of water, and then remains fasting for an additional six hours [120]. Capsule localization is determined as follows: (1) colonic entry is identified by a sustained drop in pH of more than one unit for at least five minutes, indicating passage through the ileocecal valve into the cecum; (2) capsule exit is confirmed by (i) a sudden temperature drop with loss of pressure signal, (ii) absence of the capsule on serial abdominal radiographs, or (iii) retrieval of the capsule in stool. Colonic transit time (CTT) is defined as the interval from cecal entry to expulsion. This method is noninvasive, objective, and reproducible, providing a valuable diagnostic tool for gastrointestinal motility disorders.

This ambulatory test measures transit times throughout the entire gastrointestinal tract, showing high sensitivity and specificity for confirming a diagnosis of STC and differentiating it from other motility disorders.

This approach avoids radiation exposure from repeated imaging and does not require prolonged hospitalization. However, the relatively large capsule size may pose swallowing difficulties for some patients, and the need for specialized equipment contributes to higher procedural costs.

#### Advantages of magnetic resonance imaging

Magnetic resonance imaging (MRI), with its noninvasive and radiation-free attributes, has demonstrated significant clinical value in the diagnostic evaluation of STC. This technique exploits the spin properties of hydrogen protons in an external magnetic field, utilizing precisely controlled gradient fields and radiofrequency pulse sequences to generate high-resolution images with superior soft tissue contrast. In gastrointestinal functional assessment, MRI not only delineates the anatomical layers of the bowel wall (including mucosa, muscularis, and serosa) [121], but also enables real-time observation of bowel peristalsis and direct visualization of intraluminal content transit through dynamic scanning [122, 123]. By monitoring colonic morphological changes, contraction sequences, and fecal bolus movement at different time points, MRI allows for precise evaluation of colonic transit function, providing critical imaging evidence for elucidating the pathophysiological mechanisms of STC. In STC patients, MRI typically reveals colonic dilatation, mural thickening, and markedly reduced transit velocity [124], serving as compelling diagnostic evidence. Furthermore, MRI is suitable for disease assessment in radiation-sensitive populations (e.g., pregnant women), although it is not considered essential for STC diagnosis in pregnancy [125, 126]. Thus, MRI holds unique value in the diagnosis of functional bowel disorders.

MRI provides excellent anatomical detail without ionizing radiation, offering high sensitivity and specificity for ruling out structural lesions and evaluating defecatory disorders, but it has limited sensitivity for isolated STC.

Nevertheless, MRI is limited by its relatively high cost, restricting widespread adoption in primary care settings. The prolonged scan duration necessitates sustained patient immobility, which may be poorly tolerated by elderly, frail, or claustrophobic individuals, increasing the risk of motion artifacts and compromising image quality and diagnostic accuracy [127–132]. Consequently, MRI is not considered a first-line diagnostic modality for STC.

### Laboratory diagnostic markers

Laboratory testing plays an indispensable adjunctive role in the diagnostic algorithm for STC. Precise analysis of blood and stool samples facilitates exclusion of systemic diseases and intestinal dysbiosis, providing critical clues for definitive diagnosis and individualized therapeutic planning.

#### Blood marker screening

Blood testing encompasses a range of key parameters, among which thyroid function, blood glucose, and serum calcium are particularly pertinent to STC diagnosis. Thyroid hormones exert broad regulatory effects on metabolism; in hypothyroidism, insufficient thyroxine secretion leads to glycosaminoglycan accumulation in the submucosa or smooth muscle of the intestine [58], reduced cellular metabolism, impaired smooth muscle contractility, and diminished peristalsis, culminating in STC [133]. Measurement of serum thyroid-stimulating hormone (TSH) and free thyroxine (FT4) enables accurate assessment of thyroid status; elevated TSH and reduced FT4 are indicative of hypothyroidism, providing a robust etiological basis for STC.

The association between glycemic abnormalities and STC is also significant [134]. Chronic hyperglycemia in diabetes mellitus precipitates a spectrum of metabolic derangements, including neuropathy [135] and microangiopathy [136]. Hyperglycemia induces neural degeneration and demyelination, impairing autonomic regulation of the gut, while microvascular disease results in mucosal ischemia and hypoxia, compromising enteric cellular function and motility. Studies indicate that approximately 31.2% of diabetic patients experience constipation [137]. Assessment of fasting glucose, postprandial glucose, and glycated hemoglobin (HbA1c) facilitates identification of latent diabetes risk and timely intervention to alleviate STC symptoms.

Fluctuations in serum calcium also exert significant effects on intestinal function. Calcium influences gastrointestinal health by enhancing peristalsis, softening stool, regulating fluid and electrolyte balance, and modulating inflammatory processes [138]. Hypercalcemia, as seen in hyperparathyroidism, reduces neuromuscular excitability and impairs smooth muscle contractility, leading to constipation [139]. Conversely, hypocalcemia increases smooth muscle excitability, but insufficient calcium impairs excitation–contraction coupling, also resulting in dysmotility. Measurement of serum calcium, phosphate, and parathyroid hormone (PTH) assists in excluding calcium-phosphorus metabolic disorders as confounding or causative factors in STC.

#### Significance of stool testing

Stool analysis holds unique value in STC diagnosis. Fecal microbiota profiling reflects the intestinal microecological environment, inflammatory status, and potential organic pathology. The gut microbiota, as the “invisible guardian” of intestinal health, is closely linked to the pathogenesis and progression of STC [140]. In STC patients, dysbiosis—characterized by reduced beneficial and increased pathogenic bacteria—disrupts normal metabolism, immune modulation, and neural signaling, thereby impairing colonic motility [134]. Comprehensive analysis of microbial abundance, composition, and metabolic byproducts provides insight into the intestinal microecology, guiding targeted probiotic supplementation and microbiota modulation.

In summary, laboratory diagnostic markers are intimately associated with STC diagnosis, providing complementary and corroborative information from multiple perspectives to facilitate accurate diagnosis and inform subsequent therapeutic strategies.

## Multidimensional exploration of therapeutic strategies

The management of slow transit constipation follows a stepped approach across a spectrum from conservative to surgical strategies. These multimodal treatment options are summarized in Table 1, which serves as a framework for the subsequent detailed discussion.

**Table 1:** Summary of multimodal treatment options for slow transit constipation

Treatment Category	Specific Modality	Agents(Examples)	Primary Mechanism of Action	Key Considerations
Conservative Management	Dietary Modification	Increased dietary fiber (20–25 g/day), Adequate hydration (1.5–2 L/day)	Increases stool bulk and softness; stimulates peristalsis	Foundation of therapy; requires long-term adherence
	Physical Rehabilitation	Regular toileting training, Aerobic exercise (e.g., walking, jogging, swimming), Yoga	Strengthens abdominal/pelvic muscles; stimulates colonic motility	Encourages routine and improves overall muscle function
	Psychological Intervention	Relaxation training, Cognitive-behavioral therapy, Professional psychological counseling, Biofeedback therapy	Reduces stress/anxiety; modulates gut-brain axis; retrains pelvic floor	Crucial for patients with comorbid psychological distress
	Pharmacological Therapy	Bulk-Forming Agents (e.g., Psyllium, Methylcellulose)	Absorbs water in the intestine to form soft fecal mass and stimulate peristalsis	First-line; mimics natural dietary fiber; requires adequate fluid intake
		Osmotic Laxatives (e.g., PEG, Lactulose)	Draws water into bowel lumen to soften stool	First-line; well-tolerated for long-term use
		Stimulant Laxatives (e.g., Senna, Bisacodyl)	Directly stimulates enteric nerves to induce contraction	Short-term use; risk of electrolyte imbalance with chronic use
		Secretagogues (e.g., Lubiprostone, Linaclotide)	Increases intestinal fluid secretion	Prescription agents for chronic idiopathic constipation
		Peripherally Acting μ-Opioid Receptor Antagonists (PAMORAs) (e.g., Methylnaltrexone, Alvimopan, Naloxegol)	Antagonizes opioid effects on gastrointestinal tract without compromising central analgesia	Specifically for Opioid-Induced Constipation; low systemic absorption
		Prokinetic agents (e.g., Prucalopride)	Enhances acetylcholine release, promoting colonic peristalsis	High selectivity and favorable safety profile; effective prokinetic
		Microbial Preparations (Probiotics, FMT)	Modulates gut microbiota; improves microecology and motility	Emerging evidence supports its role in restoring gut balance
Surgical Intervention	Colectomy with Ileorectal Anastomosis	Total Colectomy	Removes the entire colon to eliminate the dysfunctional segment	Considered gold standard for refractory STC; risk of diarrhea/incontinence
		Subtotal Colectomy	Preserves ileocecal region; may reduce postoperative diarrhea	Balances efficacy with lower risk of certain complications compared to total colectomy
	Segmental Colectomy	Left or Right Hemicolectomy	Resects specific colonic segment with proven delayed transit	Preserves more healthy colon; requires precise preoperative localization

*Abbreviations:* *STC* Slow Transit Constipation, *PEG* Polyethylene Glycol, *FMT* Fecal Microbiota Transplantation, *PAMORAs* Peripherally Acting μ-Opioid Receptor Antagonists

### Comprehensive approaches to conservative management

Conservative management remains the cornerstone of treatment for slow transit constipation (STC), encompassing dietary, physical, psychological, and pharmacological interventions. The primary objective is to modulate intestinal function, alleviate constipation symptoms, and enhance patient quality of life through integrated strategies.

#### Dietary modification strategies

Dietary modification constitutes the foundation of conservative therapy. An optimized dietary structure provides a favorable environment for colonic transit and facilitates fecal evacuation. Increased dietary fiber intake has been shown to significantly accelerate colonic transit time, increase defecation frequency, and augment populations of beneficial gut microbiota, thereby ameliorating clinical symptoms in STC patients [141, 142]. It is generally recommended that patients consume 20–25 g of dietary fiber daily [143], achievable through whole grains (e.g., whole wheat bread, oatmeal, brown rice), legumes (e.g., black beans, red beans), vegetables (e.g., broccoli, carrots, spinach), and fruits (e.g., kiwifruit [144], apples, bananas, pears). For instance, a daily regimen might include a bowl of oatmeal for breakfast, a black bean salad at lunch, and a serving of stir-fried vegetables at dinner, ensuring both adequate fiber intake and dietary diversity.

In addition to fiber, insufficient hydration is significantly associated with increased constipation risk [145]. Adequate water intake lubricates the intestinal lumen and facilitates stool passage. Patients are advised to consume 1,500–2,000 mL of water daily, in conjunction with fiber supplementation [146], which can markedly increase defecation frequency. Water should be ingested in divided doses to avoid renal overload. Drinking a glass of warm water upon waking may stimulate intestinal peristalsis and initiate the defecation reflex; carrying a water bottle and drinking at regular intervals throughout the day helps maintain optimal intestinal hydration.

Dietary adjustments should also include avoidance of spicy, greasy, and irritant foods, which may exacerbate intestinal dryness and worsen constipation. Regular meal timing, avoidance of overeating or prolonged fasting, and maintenance of normal digestive rhythms are recommended.

#### Physical rehabilitation guidance

Regular toileting training and effective defecation guidance are crucial for STC prevention [147]. Patients should be encouraged to establish a routine of at least two bowel movements daily, ideally upon waking or approximately 30 min postprandially. During defecation attempts, patients should be instructed to exert 50–70% of maximal effort for no more than five minutes [148].

Appropriate physical activity significantly improves intestinal function in STC patients [149, 150]. Exercise stimulates peristalsis, strengthens abdominal and pelvic floor musculature, shortens colonic transit time, and promotes fecal evacuation [151]. Aerobic activities such as walking, jogging, and swimming are recommended at a frequency of 3–5 times per week, with each session lasting at least 30 min. Walking, particularly after meals, is convenient and effective for promoting digestion and relieving constipation. Jogging enhances cardiopulmonary function and engages systemic musculature, thereby supporting intestinal motility. Swimming, by leveraging buoyancy and resistance, provides comprehensive muscular training and has a pronounced effect on intestinal function.

Additionally, exercises such as yoga [152–154], which emphasize flexibility and core strength, are beneficial for STC patients. Twisting postures (e.g., spinal twists) massage visceral organs and stimulate peristalsis, while poses such as cat-cow mobilize the spine, relax back muscles, improve abdominal circulation, and enhance intestinal vitality. Patients should select exercise modalities suited to their physical condition and preferences, maintaining long-term adherence to achieve optimal outcomes.

#### Psychological interventions

Psychological factors play a pivotal role in the pathogenesis and progression of STC, making psychological intervention an essential component of conservative management, often in conjunction with surgical or other therapeutic modalities for enhanced efficacy [155]. Chronic constipation frequently induces anxiety and depression, which in turn exacerbate intestinal dysmotility, creating a vicious cycle [156–158]. Relaxation training, including deep breathing and progressive muscle relaxation, effectively reduces psychological stress and modulates autonomic nervous system function, thereby promoting intestinal motility. Patients are advised to perform 10–15 min of deep breathing exercises each morning and evening in a quiet, comfortable environment, focusing on diaphragmatic expansion and gradual exhalation to achieve relaxation. Progressive muscle relaxation involves sequential contraction and relaxation of muscle groups from the feet upward, aiding in the recognition and alleviation of muscular tension.

Professional psychological counseling is also critical. Counselors can identify the root causes of psychological distress and provide targeted support and coping strategies. For patients whose STC is precipitated by occupational stress and anxiety, counselors may assist in developing balanced work-life plans and effective stress management techniques, such as time management and emotional regulation, thereby reducing psychological burden and improving constipation symptoms. Cognitive-behavioral therapy, by modifying maladaptive cognitions and coping mechanisms, fosters a positive outlook, enhances treatment confidence, and disrupts the psychophysiological cycle of constipation.

Biofeedback therapy, a behavioral intervention, has demonstrated efficacy in alleviating symptoms in select STC patients, improving psychological status and quality of life [159–163].

#### Advances in pharmacological therapy

Pharmacological intervention is pivotal in the conservative management of STC. Ongoing research has yielded novel agents, expanding therapeutic options. Traditional laxatives include bulk-forming agents (e.g., psyllium, methylcellulose), osmotic agents (e.g., polyethylene glycol, lactulose), stimulant laxatives (e.g., senna, bisacodyl), and secretagogues (e.g., lubiprostone, linaclotide) [164, 165], each promoting defecation via distinct mechanisms. Bulk-forming agents absorb water in the intestine, forming soft fecal masses that stimulate peristalsis; osmotic agents increase intraluminal osmotic pressure, drawing water into the bowel and softening stool; stimulant laxatives directly activate enteric nerves to induce contractions, though chronic use may result in enteric neuropathy and electrolyte disturbances, necessitating caution; secretagogues modulate intestinal ion and water secretion, increasing stool volume and frequency while improving consistency [166].

Peripherally acting μ-opioid receptor antagonists (e.g., methylnaltrexone, alvimopan, naloxegol) are effective for opioid-induced constipation. These agents exhibit low systemic absorption and do not cross the blood–brain barrier, selectively antagonizing opioid effects on the gastrointestinal tract without compromising central analgesia. They possess prokinetic potential and are effective in preventing or alleviating constipation and postoperative ileus [167].

Prokinetic agents, acting at neuromuscular junctions or specific receptors, enhance gastrointestinal motility with high selectivity and favorable cardiovascular safety profiles [168]. Prucalopride, a highly selective 5-HT4 receptor agonist, stimulates acetylcholine release from enteric neurons, promoting colonic peristalsis [169]. Multiple clinical trials have demonstrated its efficacy in improving defecation frequency and stool characteristics in STC patients, with a favorable safety profile [170–173]. Mosapride and similar agents act via analogous mechanisms, providing additional therapeutic options.

Microbial preparations have garnered increasing attention. Probiotics modulate gut microbiota composition, improve intestinal microecology, promote short-chain fatty acid production, maintain barrier integrity, and regulate gut hormone secretion, thereby alleviating constipation [174]. Bifidobacterium quadruple viable tablets, Bacillus subtilis dual viable enteric capsules, and fecal microbiota transplantation can replenish beneficial flora, suppress pathogenic bacteria, enhance barrier function, and stimulate peristalsis. Studies indicate that STC patients exhibit dysbiosis, and supplementation with microbial preparations or fecal microbiota transplantation increases microbial diversity and relieves constipation symptoms [175–179].

Pharmacological regimens should be individualized based on patient-specific clinical status, comorbidities, and drug tolerability, adhering to principles of personalized and standardized therapy. Regular assessment of therapeutic efficacy and timely adjustment of treatment protocols are essential to achieve optimal outcomes.

### Precision in surgical decision-making

For patients with refractory slow transit constipation (STC) unresponsive to pharmacological interventions, surgical management constitutes a pivotal therapeutic option. However, the selection of surgical intervention necessitates precise delineation of surgical indications, comprehensive analysis of the advantages and disadvantages of various operative techniques, and rigorous perioperative management to ensure procedural safety and efficacy, thereby optimizing patient outcomes.

#### Defining surgical indications

Surgical intervention may be considered when constipation symptoms in STC patients reach a threshold of severity, particularly after the failure of standardized conservative therapies. While the majority of patients achieve satisfactory outcomes postoperatively, a subset continues to experience complications such as diarrhea, incontinence, abdominal pain, recurrent constipation, and abdominal distension [180]. Thus, meticulous assessment and strict adherence to surgical indications by colorectal surgeons are critical determinants of surgical success [181]. Patients must be thoroughly counseled regarding operative risks and the potential for symptom recurrence, and advised to approach surgical intervention with caution [182].

Surgical treatment should be contemplated under the following conditions: First, the patient must have undergone comprehensive conservative management—including dietary modification, physical rehabilitation, psychological intervention, and adequate pharmacotherapy—for a minimum duration of six months, without meaningful symptom relief and with significant impairment of quality of life. This implies that, despite strict adherence to medical advice and multiple conservative modalities, the patient continues to suffer from persistent abdominal distension, pain, and defecatory difficulty, with substantial detriment to daily functioning, occupational performance, and psychological well-being.

Colonic transit studies serve as a critical diagnostic adjunct. If, following oral administration of radiopaque markers, more than 80% of markers remain within the colon after 72 h or longer, and repeated assessments have excluded false positives due to laxative use or anatomical anomalies, severe colonic transit dysfunction is strongly suggested, thereby increasing the necessity for surgical intervention. In certain cases, colonic transit time may extend to several weeks, with fecal matter virtually “stagnant” within the colon; such patients are more likely to benefit from surgical management.

Exclusion of alternative etiologies for constipation is equally essential, including systemic diseases such as hypothyroidism, diabetic neuropathy, pelvic floor dysfunction, or local structural abnormalities. Comprehensive laboratory evaluation, imaging, and pelvic floor function testing are required to confirm that constipation is primarily attributable to delayed colonic transit, rather than confounding factors. For example, in hypothyroid patients, surgical intervention targeting constipation alone, without correction of thyroid dysfunction, is likely to result in recurrence, as the underlying motility disorder remains unaddressed. Only after thorough assessment and confirmation that the patient meets these stringent criteria should surgery be considered a viable and effective option for alleviating chronic constipation.

#### Analysis of common surgical procedures

The choice of surgical technique is another critical determinant of therapeutic success. Multiple operative modalities are available for STC, with the most prevalent being total colectomy with ileorectal anastomosis, subtotal colectomy, segmental colectomy, and colonic exclusion procedures [183, 184]. The first two approaches are most widely utilized and have demonstrated efficacy in improving quality of life in STC patients [185, 186].

Given the distinct advantages and limitations of each procedure, individualized selection based on patient-specific factors is imperative. Total colectomy with ileorectal anastomosis is regarded as the gold standard for refractory STC [187–189]. With advances in minimally invasive surgery, laparoscopic techniques [190, 191], as well as natural orifice specimen extraction and intracorporeal anastomosis [192], have been increasingly adopted. This procedure effectively ameliorates constipation and enhances quality of life for most patients; however, it is associated with significant surgical trauma, loss of colonic function, and impaired water and electrolyte absorption, leading to postoperative complications such as diarrhea, incontinence, abdominal distension, and pain in some cases [180, 181]. Therefore, careful preoperative evaluation of indications, vigilant postoperative monitoring of electrolyte balance, timely supplementation, and adjunctive antidiarrheal therapy are essential to facilitate recovery.

Subtotal colectomy, which preserves the ileocecal region and performs cecorectal anastomosis, is considered an effective strategy to mitigate complications associated with total colectomy. This approach retains partial colonic function, achieves therapeutic outcomes comparable to total colectomy with ileorectal anastomosis [193], and significantly reduces the incidence of incontinence and diarrhea [194]. However, the risk of STC recurrence is relatively higher due to the preservation of colonic segments [183].

Segmental colectomy, such as left or right hemicolectomy [195], is indicated for patients with localized colonic transit delay as demonstrated by transit studies. Resection of the affected segment can improve overall colonic transit while preserving more normal colon, thereby minimizing impact on storage and absorptive functions and reducing the risk of postoperative diarrhea compared to subtotal colectomy [181], making it a preferable option for select STC patients [196]. The limitation of this approach lies in the potential for incomplete symptom relief if preoperative localization is inaccurate, necessitating continued laxative use or, in some cases, reoperation for further resection due to persistent dysfunction in the residual colon [197].

#### Key points in perioperative management

Scientific perioperative management is fundamental to surgical success and patient recovery. Preoperatively, thorough bowel preparation is essential. Patients should commence a residue-free semi-liquid diet three days prior to surgery, transition to a liquid diet one day before, and ingest bowel-cleansing agents such as polyethylene glycol electrolyte solution with copious fluids to ensure a clear operative field and reduce intraoperative infection risk. Nutritional support is also critical, particularly for patients with malnutrition or hypoproteinemia secondary to chronic constipation; intravenous administration of albumin and amino acids may be required to optimize nutritional status and enhance surgical tolerance. Psychological counseling should not be neglected, as many patients experience preoperative anxiety and fear regarding surgical risks and postoperative recovery. Healthcare providers must offer detailed explanations of the procedure, expected outcomes, and potential complications to alleviate psychological burden and foster a positive, cooperative attitude.

Postoperatively, close monitoring of vital signs is imperative, with particular attention to abdominal pain, distension, fever, and nausea or vomiting, to promptly identify and manage complications. For patients undergoing subtotal colectomy, vigilance for postoperative small bowel obstruction is warranted, with an incidence of approximately 10%–15%. Should symptoms such as cessation of flatus and stool, severe paroxysmal abdominal pain arise, immediate conservative management—including fasting, gastrointestinal decompression, and fluid resuscitation—should be instituted, with most cases resolving without further intervention, though a minority may require reoperation. Wound care must be meticulous, maintaining incision cleanliness and dryness, with regular dressing changes to prevent infection. Rational rehabilitation is equally vital; early mobilization, including in-bed turning and limb exercises, should be encouraged to promote gastrointestinal motility and prevent thrombosis. Activity should be gradually increased according to recovery status, progressing from sitting and standing at the bedside to slow ambulation, thereby expediting restoration of gastrointestinal function and physical strength, ultimately optimizing surgical outcomes and enhancing long-term quality of life.

## Conclusion and future perspectives

### Summary of research findings

This study has provided an in-depth exploration of slow transit constipation (STC), yielding significant advances in the understanding of its pathogenesis, diagnostic modalities, and therapeutic strategies.

With respect to pathogenesis, it has been elucidated that intestinal dysmotility constitutes the central mechanism, involving smooth muscle dysfunction, interstitial cells of Cajal (ICC) pathology, and disturbances in neural regulation. Atrophy and fibrosis of smooth muscle cells, along with aberrant calcium signaling pathways, result in diminished contractile capacity. A reduction in ICC number, as well as morphological and functional abnormalities, disrupts the fundamental electrical rhythmicity of the colon. Degenerative changes within the enteric nervous system (ENS), neurotransmitter imbalances, and autonomic nervous system dysfunction collectively contribute to impaired colonic motility. Endocrine and hormonal factors play a pivotal role in STC pathogenesis; aberrant thyroid hormone secretion affects cellular metabolism and smooth muscle contraction, while fluctuations in sex hormones during various female physiological stages interfere with intestinal peristalsis. Psychological factors, through stress response mechanisms and chronic negative emotions, alter the intestinal microenvironment and neural transmission, thereby participating in the onset and progression of STC.

From a diagnostic perspective, emphasis is placed on the assessment of clinical symptoms—reduced defecation frequency, hard stools, and straining are hallmark features, often accompanied by abdominal distension and pain, which provide important diagnostic clues. Imaging modalities each offer distinct advantages: colonic transit studies accurately quantify colonic transit time; defecography effectively diagnoses pelvic floor dysfunction; and magnetic resonance imaging, with its high resolution, absence of radiation, and multiplanar capabilities, enables comprehensive evaluation of colonic motility and pelvic floor function. Nevertheless, each modality has inherent limitations. Laboratory investigations are indispensable, with blood tests excluding systemic disorders such as thyroid dysfunction, hyperglycemia, and hypercalcemia, while stool analyses reflect the intestinal microecology and inflammatory status, aiding in etiological clarification.

Therapeutic strategies are multifaceted. Conservative management encompasses dietary modification (increased fiber and fluid intake, avoidance of irritant foods), exercise rehabilitation (aerobic activity, yoga), and psychological interventions (relaxation training, counseling). Pharmacological therapy has evolved, with novel prokinetic agents and microbiota-based preparations expanding therapeutic options. Surgical intervention requires precise patient selection, with strict delineation of indications based on failure of conservative therapy, colonic transit study results, and exclusion of alternative etiologies. Each surgical technique presents distinct advantages and limitations: subtotal colectomy demonstrates robust efficacy but is associated with significant morbidity, while segmental colectomy offers targeted intervention but carries a risk of inaccurate localization. Perioperative management is critical, emphasizing thorough bowel preparation, nutritional support, and psychological counseling preoperatively, as well as vigilant monitoring of vital signs, prevention of complications, and enhanced wound and rehabilitation care postoperatively.

The diagnosis and management of STC necessitate a multidisciplinary approach, integrating the expertise of gastrointestinal surgery, gastroenterology, radiology, laboratory medicine, and psychiatry to formulate individualized, precision-based treatment regimens that optimize therapeutic outcomes and improve patient quality of life.

### Future research directions

Despite considerable progress in the field of STC, numerous critical issues remain to be addressed.

At the basic research level, genetic investigations are anticipated to provide novel insights into STC pathogenesis. Future studies should focus on identifying susceptibility genes associated with STC and elucidating the impact of genetic polymorphisms on intestinal motility, neuroendocrine regulation, and the gut microbiome. Advanced techniques such as genome-wide association studies (GWAS) will facilitate large-scale screening of genetic variants across diverse populations, enabling the construction of risk models linking genetic factors to STC susceptibility and paving the way for early genetic diagnosis and preemptive intervention. Research into cellular signaling pathways is equally crucial; further dissection of aberrant signal transduction within intestinal smooth muscle cells, ICCs, and neurons may reveal novel therapeutic targets. Evidence suggests that specific intracellular signaling pathways play a key role in regulating intestinal motility [198–201]; modulation of these pathways via selective inhibitors or activators may yield innovative therapeutic breakthroughs for STC.

In clinical practice, optimization of surgical strategies remains a priority. With the advent of precision medicine, future efforts may leverage advanced preoperative assessments—such as multimodal imaging integration and molecular markers of intestinal function—to tailor surgical interventions, enhance procedural accuracy and efficacy, and reduce postoperative complications. Long-term postoperative follow-up studies are urgently needed; the establishment of large-scale, multicenter follow-up databases will enable longitudinal assessment of bowel function recovery, quality of life, and the evolution of potential complications, thereby providing a robust foundation for comprehensive evaluation of surgical outcomes and further refinement of surgical protocols.

In the realm of conservative therapy, exploration of combination regimens holds significant promise. Detailed investigation of synergistic mechanisms among various conservative modalities—such as optimal integration of dietary modification, pharmacotherapy, psychological intervention, and exercise rehabilitation—may facilitate the development of more effective comprehensive treatment packages. Large-scale, multicenter randomized controlled trials comparing the efficacy of combination versus monotherapy approaches will provide high-level evidence to inform clinical practice, enabling patients to derive maximal benefit from individualized, precision-based combination therapies and improving long-term prognosis.

The future of STC research is replete with both challenges and opportunities. Interdisciplinary collaboration and the integration of basic and clinical research will be essential to overcoming this complex disorder, ultimately enhancing patient quality of life and alleviating the societal healthcare burden.

## Data Availability

Data sharing is not applicable to this article as no new data were created or analyzed in this study. As a narrative review, all information and conclusions are based on previously published studies which are cited in the reference list.
